# Atrial Secondary Mitral Regurgitation: Pathophysiology, Diagnosis, and Surgical Implications

**DOI:** 10.3390/medicina62030520

**Published:** 2026-03-11

**Authors:** Damiano Venturiello, Giuseppe Campolongo, Emiliano Marco Navarra, Giuseppe Speziale

**Affiliations:** 1 Department of Cardiac Surgery, San Carlo di Nancy Hospital, GVM Care & Research, 00165 Rome, Italy; gcampolongo@gvmnet.it (G.C.); enavarra@gvmnet.it (E.M.N.); gspeziale@gvmnet.it (G.S.); 2 Department of Cardiac Surgery, Anthea Hospital and Santa Maria Hospital, GVM Care & Research, 70124 Bari, Italy

**Keywords:** atrial secondary mitral regurgitation, atrial functional mitral regurgitation, mitral annuloplasty, mitral valve repair, atrial fibrillation, left atrial remodeling, echocardiography, cardiac magnetic resonance, transcatheter edge-to-edge repair, heart failure with preserved ejection fraction, surgical ablation

## Abstract

*Background and Objectives:* Atrial secondary mitral regurgitation (A-SMR), also referred to as atrial functional mitral regurgitation, has emerged as a distinct clinical phenotype characterized by left atrial enlargement, mitral annular dilatation, and preserved left ventricular geometry and systolic function. Frequently associated with long-standing atrial fibrillation (AF) and heart failure with preserved ejection fraction (HFpEF), A-SMR challenges the traditional ventricular-centered classification of functional mitral regurgitation (MR) and is increasingly recognized as a clinically relevant condition. *Materials and Methods:* This narrative review provides an updated and critical overview of current evidence on A-SMR. We summarize available data on pathophysiology, diagnostic imaging, natural history, and therapeutic strategies, with particular emphasis on implications for cardiac surgery and clinical decision-making. Evidence was derived from observational studies, registry analyses, interventional reports, and contemporary guideline documents. *Results:* A-SMR is primarily driven by atrial remodeling and annular dilatation, with minimal contribution from ventricular distortion or leaflet tethering. Echocardiography and Magnetic Resonance Imaging (MRI) play a central role in diagnosis and phenotypic characterization, allowing differentiation from ventricular functional MR and identification of distinct A-SMR subtypes with potential therapeutic implications. A-SMR is a progressive condition associated with worsening symptoms and adverse clinical outcomes. Rhythm control strategies may reduce MR severity in selected patients by promoting atrial reverse remodeling. Transcatheter edge-to-edge repair (TEER) represents a treatment option for selected high-risk patients, although concerns regarding long-term durability remain in this predominantly annular disease. From a pathophysiological standpoint, surgical mitral valve repair based on annuloplasty directly targets the dominant mechanism of A-SMR and has been associated with favorable outcomes in appropriately selected patients. *Conclusions:* A-SMR is a distinct and increasingly recognized form of functional MR requiring a mechanism-oriented diagnostic and therapeutic approach. The 2025 ESC/EACTS Guidelines for the management of valvular heart disease have acknowledged A-SMR as a specific clinical phenotype, although dedicated phenotype-specific management recommendations remain limited. Surgical mitral valve repair, particularly when combined with AF ablation, represents a rational treatment strategy in selected patients and may improve long-term outcomes.

## 1. Introduction

Atrial secondary mitral regurgitation, also termed atrial functional mitral regurgitation, has been recognized as a distinct form of secondary MR occurring in the setting of atrial remodeling and HFpEF [1]. Large-scale registry data from the National Echocardiographic Database of Australia have shown that A-SMR accounts for approximately 40% of cases of moderate-to-severe functional MR, predominantly affecting elderly women with AF and carrying a substantial long-term mortality risk [2].

Recent large-scale echocardiographic investigations have highlighted the marked heterogeneity of A-SMR, demonstrating that atrial, ventricular, and combined (dual) phenotypes frequently coexist and exhibit distinct patterns of structural remodeling, clinical progression, and prognosis, thereby underscoring the need for a refined pathophysiological classification of secondary MR [3]. Detailed imaging analyses have further refined the concept of A-SMR by identifying mechanistic subtypes based on leaflet motion and annular–atrial interaction, with emerging evidence suggesting that these phenotypes may differ not only in structural characteristics but also in response to transcatheter therapies and clinical outcomes [4].

The 2025 ESC/EACTS Guidelines for the management of valvular heart disease formally recognize A-SMR as a distinct entity within secondary MR, providing dedicated diagnostic criteria and specific management considerations that differentiate atrial-driven from ventricular-driven disease [5]. Restoration of sinus rhythm has been shown to induce biatrial and ventricular reverse remodeling and to reduce the severity of A-SMR, supporting a causal relationship between AF–related structural remodeling and valvular incompetence [6]. Registry data from the international EuroSMR cohort indicate that A-SMR represents nearly 8% of patients undergoing TEER, with acceptable procedural success and mid-term survival, although right ventricular dysfunction emerges as a key determinant of prognosis in this population [7]. Surgical series focusing on isolated A-SMR have further demonstrated that annuloplasty-based mitral valve repair provides durable correction with low recurrence rates and favorable long-term survival, reinforcing the concept that not all forms of functional MR share identical mechanisms or therapeutic implications [8].

However, although the distinction between atrial and ventricular forms of secondary MR has gained increasing recognition, its implications for classification, disease progression, and optimal therapeutic strategy remain incompletely defined.

Functional MR has traditionally been regarded as a consequence of left ventricular remodeling and systolic dysfunction. In this classical framework, mitral valve incompetence develops secondary to ventricular dilatation, papillary muscle displacement, and leaflet tethering, while the valve leaflets themselves remain structurally normal [9]. This ventricular-centered paradigm has long guided both diagnostic classification and therapeutic strategies in patients with secondary MR.

In recent years, however, growing clinical and echocardiographic evidence has challenged this concept by identifying a distinct form of functional MR occurring in the presence of preserved left ventricular size and systolic function. In this setting, MR is primarily driven by left atrial enlargement and mitral annular dilatation rather than by ventricular distortion [10]. This entity, commonly referred to as atrial secondary mitral regurgitation (A-SMR) or atrial functional mitral regurgitation, represents a paradigm shift in the understanding of functional mitral valve disease and differs from the ventricular secondary mitral regurgitation (V-SMR).

A-SMR is most frequently observed in patients with long-standing AF and in those with HFpEF. These conditions are characterized by chronically elevated left atrial pressure and volume, which promote progressive atrial remodeling and annular enlargement over time [11]. As atrial size increases, leaflet coaptation becomes insufficient despite the absence of primary leaflet pathology, leading to clinically significant MR.

Epidemiological data on A-SMR remain limited, as this entity has only recently been recognized as a distinct phenotype of functional MR. In a retrospective cohort study including 378 consecutive patients with moderate-to-severe or severe functional MR, Okamoto et al. identified 90 patients (24%) with A-SMR based on preserved or mildly reduced left ventricular systolic function and significant left atrial remodeling [12]. In a large unselected cohort, Chen et al. showed that although atrial functional MR accounted for approximately 39% of cases and purely ventricular functional MR for about 14%, nearly 50% of patients exhibited a significant ventricular contribution to MR when combined atrial–ventricular (dual) phenotypes were considered [3]. The dual functional MR phenotype refers to a mixed form of functional MR in which atrial-driven mechanisms (left atrial enlargement and annular dilatation) coexist with ventricular-driven mechanisms (left ventricular remodeling and leaflet tethering) [3]. This phenotype likely represents an advanced or progressive stage of disease, in which initial atrial dysfunction is accompanied over time by ventricular structural changes [3]. Importantly, A-SMR should not be considered a benign entity. Okamoto et al. reported that among the 90 patients classified as atrial functional MR, all-cause mortality occurred in 16% (vs. 29% in V-SMR) and heart failure (HF) hospitalization in 36% (vs. 50% in V-SMR) during a median follow-up of 4.1 years [12].

A-SMR differs from V-SMR not only in terms of pathophysiology but also with respect to patterns of cardiac remodeling and disease progression. In a comparative cohort analysis, atrial and ventricular functional MR were associated with distinct structural and clinical phenotypes, with atrial-driven disease exhibiting a stronger relationship with left atrial size, rhythm status, and annular geometry [13]. These differences suggest that therapeutic strategies effective in V-SMR may not be directly applicable to patients with A-SMR.

Despite its growing recognition, A-SMR remains insufficiently addressed in current clinical guidelines, without providing specific recommendations tailored to atrial-driven disease [5,14]. This lack of dedicated guidance contributes to uncertainty in clinical practice, particularly when choosing among medical therapy, rhythm control strategies, transcatheter interventions, and surgical repair.

Given these considerations, a comprehensive and mechanism-oriented evaluation of A-SMR is essential. Improved understanding of its pathophysiology, imaging characteristics, natural history, and response to different therapeutic approaches may help refine patient selection and optimize outcomes. In this context, cardiac surgery plays a pivotal role, as surgical annuloplasty directly targets the dominant anatomical substrate of A-SMR and offers the opportunity for concomitant treatment of AF [15].

**Figure 1:**
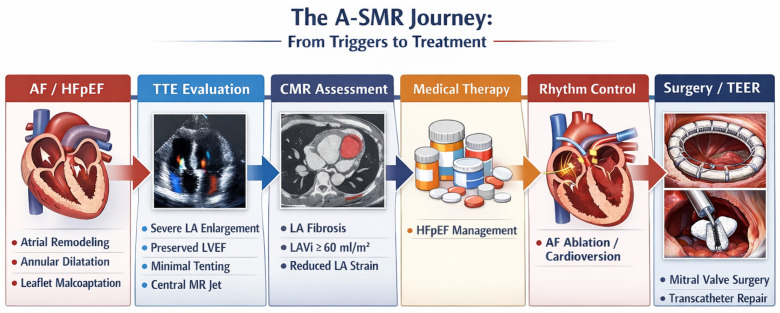
The A-SMR Journey. Abbreviations: AF, Atrial Fibrillation; A-SMR, Atrial Secondary Mitral Regurgitation; CMR, Cardiac Magnetic Resonance; HFpEF, Heart Failure with preserved Ejection Fraction; LA, Left Atrium; LAVi, Left Atrial Volume Index; LVEF, Left Ventricle Ejection Fraction; MR, Mitral Regurgitation; TEER, Transcatheter Edge-to-Edge Repair; TTE, Transthoracic Echocardiography.

Figure 1 provides an overview of the pathophysiological cascade, as well as the diagnostic and management pathways in A-SMR.

## 2. Methodology and Study Selection

This narrative review summarizes current evidence on A-SMR with a focus on diagnostic definitions, imaging assessment, clinical outcomes, and implications for cardiac surgery. A targeted literature search was performed in PubMed/MEDLINE and Scopus from inception to December 2025 using combinations of the terms “atrial functional mitral regurgitation”, “atrial secondary mitral regurgitation”, “mitral annulus”, “atrial fibrillation”, “annuloplasty”, “mitral valve repair”, and “transcatheter edge-to-edge repair”. Additional records were identified by manual screening of reference lists of relevant articles. We prioritized English-language studies in adult populations, including observational cohorts, registry studies, interventional reports, meta-analyses, and contemporary reviews. Case reports were considered only when providing unique mechanistic or surgical insights. Given the narrative design, no formal risk-of-bias assessment was performed; however, evidence was critically appraised with attention to phenotype definition, imaging criteria, and outcome ascertainment. Overall, 72 unique articles were included: 26 original (non-registry) clinical/imaging studies, 5 registry/database analyses, 15 surgical series, 4 randomized controlled trials, 1 meta-analysis, 16 review/scoping/expert opinion articles, 3 consensus/viewpoint documents, and 2 guideline documents.

## 3. Pathophysiology of Atrial Secondary Mitral Regurgitation

**Figure 2:**
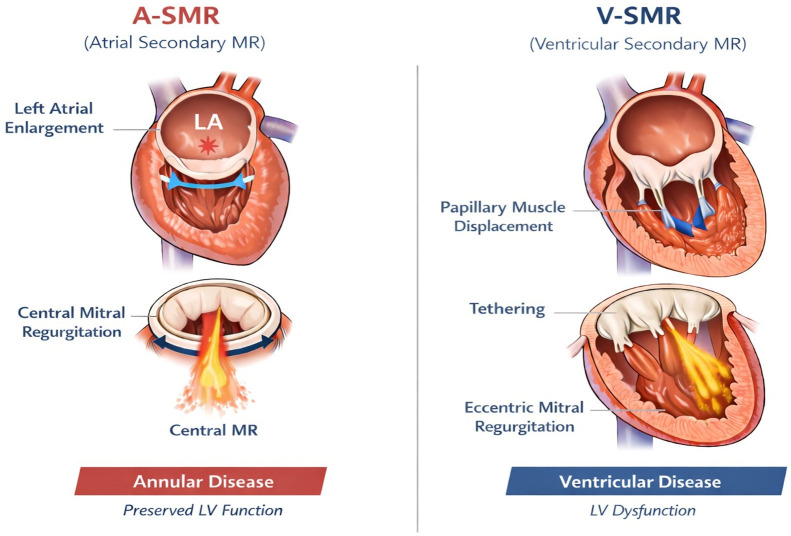
Pathophysiology of A-SMR and V-SMR. In the A-SMR panel, the red asterisk denotes left atrial enlargement. The light blue bidirectional arrow highlights circumferential mitral annular expansion, wheras the dark blue bidirectional arrow indicates annular dilatation (increased anteroposterior annular dimension). Together, these annular changes reduce leaflet coaptation and result in central mitral regurgitation despite preserved left ventricular function. Abbreviations: A-SMR, Atrial Secondary Mitral Regurgitation; LA, Left Atrium; LV, Left Ventricle; MR, Mitral Regurgitation; V-SMR, Ventricular Secondary Mitral Regurgitation.

A-SMR is fundamentally an atrial-driven disease. In contrast to V-SMR, in which mitral valve incompetence arises from left ventricular remodeling and papillary muscle displacement, A-SMR develops in the setting of preserved ventricular geometry and systolic function, with regurgitation primarily resulting from atrial and annular remodeling [16] (Figure 2).

### 3.1. Left Atrial Remodeling and Annular Dilatation

Chronic elevation of left atrial pressure and volume represents the central trigger for structural remodeling in A-SMR. Long-standing AF and diastolic dysfunction lead to progressive atrial enlargement, wall stretch, and fibrosis, which in turn directly affect mitral annular size and geometry [17]. Because the mitral annulus is anatomically coupled to the left atrium (LA), atrial dilatation translates into annular enlargement even in the absence of ventricular dilatation.

Annular dilatation in A-SMR predominantly involves the posterior and lateral segments of the annulus, resulting in a more circular and flattened configuration. This geometric alteration reduces the coaptation surface between the mitral leaflets despite preserved leaflet structure and mobility, thereby generating functional regurgitation [18].

Although AF is a central driver of A-SMR, its underlying aetiology and the structural stage of atrial cardiomyopathy may influence the degree and reversibility of atrial remodeling. Contemporary consensus documents recognize atrial cardiomyopathy as a spectrum of structural, architectural, and fibrotic alterations with heterogeneous triggers, including inflammatory, metabolic, hypertensive, and HF–related mechanisms [19]. In selected cases, potentially reversible substrates and modifiable drivers may respond to targeted interventions—such as optimization of cardiovascular (CV) risk factors, RAAS inhibition, or rhythm-control strategies—resulting in partial atrial reverse remodeling [20].

However, the reversibility of atrial remodeling depends on both the duration and the biological substrate of the underlying injury. Early or predominantly functional remodeling—characterized by atrial dilatation with limited structural alteration—may be at least partially reversible following rhythm control or optimization of loading conditions. In contrast, chronic remodeling marked by advanced fibrosis, extracellular matrix expansion, and persistent atrial dilatation is generally associated with limited or absent reversibility, as structural alterations become maladaptive and self-perpetuating [20,21]. In patients with A-SMR, the extent of atrial fibrosis has been shown to influence the response to rhythm-control strategies, with advanced fibrotic remodeling predicting reduced efficacy of catheter ablation and a lower likelihood of sustained reverse remodeling [21]. Consequently, in advanced stages of atrial cardiomyopathy, etiological therapy alone is unlikely to restore durable mitral valve competence. In these cases, a mechanism-oriented management strategy is required, which may include rhythm-control interventions, surgical annuloplasty, or TEER according to clinical status and anatomical characteristics [21]. Therefore, although the identification of potentially reversible substrates remains clinically important and may guide upstream therapeutic strategies, it does not obviate the need for dedicated valve-oriented treatment in patients with advanced structural remodeling and established A-SMR.

### 3.2. Leaflet–Annulus Mismatch

Mitral leaflets may undergo adaptive enlargement in response to chronic loading conditions; however, in many patients with A-SMR, this compensatory mechanism is insufficient to match the degree of annular dilatation. The resulting leaflet–annulus mismatch represents a key mechanism underlying atrial-driven MR [21]. Unlike V-SMR, leaflet tethering and subvalvular distortion are typically minimal, and coaptation failure occurs predominantly at the annular level.

This mechanistic distinction has important therapeutic implications, as interventions aimed at restoring annular size and geometry directly address the primary cause of regurgitation in A-SMR.

## 4. Imaging Assessment and Diagnostic Criteria

Imaging plays a central role in the diagnosis and management of A-SMR. Because A-SMR is defined by preserved left ventricular geometry and the absence of primary mitral valve disease, a comprehensive imaging approach is essential to correctly identify the atrial-driven mechanism of regurgitation and to distinguish it from V-SMR [22].

### 4.1. Standard Echocardiographic Evaluation

Transthoracic echocardiography (TTE) represents the first-line imaging modality for patients with suspected A-SMR. A systematic evaluation should document preserved left ventricular size and systolic function, exclude primary leaflet abnormalities, and assess the severity of MR. Marked left atrial enlargement is a hallmark finding and should prompt careful evaluation of annular dimensions and valve coaptation [22].

MR jets in A-SMR are typically central or mildly posteriorly directed, reflecting symmetric annular dilatation rather than asymmetric leaflet tethering, which is more common in V-SMR [22].

Transesophageal echocardiography (TEE) may be useful to exclude subtle leaflet pathology and to better define annular anatomy, particularly in patients being considered for interventional or surgical treatment [22].

### 4.2. Mechanistic and Geometric Parameters

Several echocardiographic parameters are particularly informative for recognizing atrial-driven MR. Measurements of mitral annular dimensions, including anteroposterior and commissural diameters, allow quantification of annular dilatation [22]. In contrast to V-SMR, indices of leaflet tethering, such as tenting height and tenting area, are typically minimal in A-SMR [23].

Assessment of leaflet length and coaptation depth provides additional insight into leaflet–annulus mismatch. In many patients with A-SMR, leaflet tissue is relatively preserved, whereas coaptation failure results from annular enlargement rather than from subvalvular distortion [22].

### 4.3. Advanced Imaging and Atrial Function

Three-dimensional echocardiography has improved the assessment of mitral annular geometry and leaflet coaptation in A-SMR. By enabling accurate reconstruction of annular shape and dynamic changes throughout the cardiac cycle, three-dimensional imaging facilitates refined phenotypic characterization and improves patient selection for intervention [24].

In addition, evaluation of left atrial function using strain imaging provides complementary information on the severity of atrial myopathy. Reduced atrial strain has been associated with advanced atrial remodeling and may help identify patients at increased risk of persistent or recurrent MR after treatment [22].

Advanced atrial remodeling represents a critical stage in the natural history of A-SMR and may identify a “point of no return” beyond which isolated correction of MR yields limited benefit. No rigid, universally validated cut-off values currently exist specifically for A-SMR, but several imaging parameters have been associated with adverse outcomes and advanced atrial myopathy. Severe left atrial enlargement—commonly defined as a left atrial volume index (LAVi) ≥ 60 mL/m^2^—has been shown to correlate with worse prognosis in MR and HF populations, reflecting longstanding volume and pressure overload [25].

Beyond chamber size, functional assessment provides incremental information. Speckle-tracking studies have demonstrated that reduced left atrial reservoir strain (typically <15%) and impaired late diastolic strain rate are markers of atrial fibrosis and mechanical dysfunction [26]. In patients with HFpEF, longitudinal atrial dysfunction has been independently associated with worse functional status and higher filling pressures, supporting the concept of a fibrotic atrial cardiomyopathy rather than purely passive remodeling. Cardiac magnetic resonance (CMR) studies further corroborate this association, demonstrating an inverse relationship between atrial strain and the degree of atrial fibrosis [27].

In the context of A-SMR, the combination of marked atrial enlargement and severely impaired atrial strain should therefore be considered a red flag for advanced disease. Early referral for surgical intervention—before extreme annular dilatation and irreversible atrial fibrosis develop—may improve the likelihood of durable repair and reverse remodeling.

Cardiac computed tomography (CT) may provide valuable mechanistic insights into the differentiation between atrial and ventricular functional MR. In a retrospective CT-based analysis of 183 patients with functional MR, atrial MR accounted for approximately 10% of cases and was predominantly characterized by left atrial enlargement and annular dilation, with relatively preserved subvalvular geometry and minimal papillary muscle displacement. Conversely, ventricular MR was associated with significant left ventricular remodeling, papillary muscle displacement, and more pronounced leaflet tethering [28]. These findings indicate that CT imaging can be a useful adjunct in clarifying the underlying mechanism of secondary MR and may support more tailored therapeutic decision-making.

For several years, the use of CMR has become increasingly widespread in the field of valvular heart disease, particularly in MR. It allows an in-depth assessment of the mitral valve apparatus, leaflet morphology, and papillary muscles, and can also precisely characterize the impact of MR on left atrial and ventricular remodeling [29].

The DECAAF study demonstrated that the extent of left atrial fibrosis, quantified by delayed-enhancement MRI, is a strong and independent marker of atrial disease severity and clinical progression in patients with AF. Increasing degrees of atrial fibrosis were associated with worse clinical outcomes and reduced response to rhythm-control strategies, reflecting advanced atrial remodeling and loss of atrial compliance [30].

Cawley et al. performed a prospective head-to-head comparison between TTE and CMR for the quantification of chronic mitral and aortic regurgitation in 57 patients studied on the same day [31]. The study demonstrated that CMR provides significantly lower intra- and interobserver variability than TTE for regurgitant volume and ventricular volume measurements, with particularly strong reproducibility for phase-contrast flow assessment of regurgitation. Although both modalities showed substantial variability in MR quantification, CMR tended to be more consistent than Doppler-based echocardiographic methods, especially when annular geometry and ventricular volumes were difficult to define. These findings highlight the limitations of echocardiography in complex or geometrically distorted valves and support the role of CMR as a complementary tool for accurate and reproducible quantification of regurgitation severity, particularly when precise serial assessment is required for clinical decision-making or timing of intervention [31].

## 5. Natural History and Clinical Impact

A-SMR is increasingly recognized as a progressive condition rather than a benign echocardiographic finding. While mild forms may initially be well tolerated, moderate-to-severe A-SMR is associated with an increased risk of HF hospitalization and mortality [12,32].

In a large retrospective cohort of 635 patients with predominantly mild-to-moderate A-SMR and preserved left ventricular function, the incidence of new-onset left ventricular systolic dysfunction was low and comparable to matched patients with no or mild MR. Progression to severe MR was uncommon (1.9 per 100 person-years), whereas regression to no/trivial MR occurred more frequently (3.9 per 100 person-years). Importantly, neither progression nor regression was independently associated with mortality, which was instead driven by markers of diastolic dysfunction and adverse ventricular remodeling [32].

As we previously said, in the study by Okamoto et al., moderate-to-severe A-SMR was instead associated with substantial event rates. Over a median follow-up of 4.1 years, all-cause mortality occurred in 16% of patients and HF hospitalization in 36%, underscoring the non-negligible clinical impact of this condition [12].

A-SMR appears to be part of a self-perpetuating cycle of atrial remodeling. HFpEF and AF promote left atrial enlargement and annular dilatation, leading to impaired leaflet coaptation and MR. In turn, regurgitation increases atrial volume overload and wall stress, further exacerbating atrial dilation and sustaining both AF and MR severity. This self-perpetuating mechanism may ultimately lead to stabilization of significant regurgitation that becomes less responsive to rhythm or medical therapies [1].

A-SMR has important hemodynamic consequences. Chronic left atrial volume overload may lead to sustained elevation of pulmonary venous pressure and the development of pulmonary hypertension [2]. In advanced stages, pulmonary hypertension may result in right ventricular dysfunction and secondary tricuspid regurgitation (TR), further complicating the clinical course and adversely affecting prognosis [24].

Timing appears to be a critical determinant of outcomes in patients with A-SMR. Earlier stages of disease, characterized by moderate annular dilatation and less advanced atrial remodeling, may retain partial reversibility, particularly when sinus rhythm can be restored [6]. In contrast, delayed intervention in the presence of severe atrial enlargement, persistent AF, and established pulmonary hypertension is associated with less favorable outcomes and a higher likelihood of residual or recurrent MR after treatment [2].

## 6. Non-Surgical Treatment Strategies

Non-surgical management of A-SMR is primarily directed toward symptom control and treatment of associated conditions rather than correction of the underlying anatomical substrate. Because A-SMR is driven by atrial remodeling and annular dilatation, medical and rhythm-based therapies may influence disease severity in selected patients but generally do not provide definitive resolution of MR [5].

### 6.1. Medical Therapy

Medical therapy in patients with A-SMR should be considered within the framework of contemporary HFpEF management, a condition that frequently coexists with atrial-driven MR. Diuretic therapy remains essential for congestion relief and symptom control by reducing left atrial pressure; however, it does not directly address mitral annular dilatation or restore leaflet coaptation and therefore has a limited impact on the structural progression of A-SMR [1]. Sodium–glucose cotransporter-2 (SGLT2) inhibitors have emerged as a foundational therapy in HFpEF, with large randomized trials, such as EMPEROR-Preserved and DELIVER, demonstrating significant reductions in HF hospitalizations and improvements in patient-reported outcomes across the spectrum of preserved and mildly reduced left ventricular ejection fraction (LVEF) [33,34].

In patients with A-SMR occurring in the HFpEF setting, SGLT2 inhibitors should therefore be regarded as part of optimized background therapy. While they do not directly modify annular geometry, their favorable effects on cardiac filling pressures and congestion may indirectly mitigate symptom burden and dynamic variability of MR severity [33,34]. Overall, pharmacological therapy in A-SMR should be viewed as supportive and prognostically beneficial, particularly for HFpEF and comorbidity management, but insufficient as a standalone strategy for correcting atrial-driven MR. Persistent moderate-to-severe A-SMR despite optimized medical therapy should prompt consideration of interventional or surgical treatment [1].

### 6.2. Rhythm and Rate Control Strategies

Given the central role of AF in the pathophysiology of A-SMR, rhythm control strategies are of particular interest. Restoration and maintenance of sinus rhythm may promote atrial reverse remodeling, improve atrial contractile function, and partially restore annular dynamics. In selected patients, particularly those with less advanced atrial remodeling and shorter duration of AF, rhythm control has been associated with a reduction in MR severity [6].

Catheter ablation represents an important rhythm control option in appropriately selected patients and may contribute to improvement in MR by reducing atrial size and pressure over time [35]. However, success rates decrease markedly in the presence of long-standing persistent AF and severe atrial enlargement, limiting the applicability of this approach in many patients with established A-SMR [36]. Rate control strategies, while important for symptom management, have a limited effect on atrial remodeling and are unlikely to substantially influence the course of atrial-driven MR [1,36].

### 6.3. Transcatheter Edge-to-Edge Repair

TEER has emerged as a therapeutic option for patients with severe A-SMR who remain symptomatic despite optimized medical and rhythm management and who are considered not eligible for surgery (Class IIb Level B Recommendation in 2025 ESC guidelines) [5].

While landmark trials such as COAPT and MITRA-FR demonstrated divergent outcomes in V-SMR populations, these studies predominantly enrolled patients with ventricular remodeling and leaflet tethering, rather than annular-dominant disease [37,38]. In A-SMR, the primary mechanism is annular dilatation with relatively preserved leaflet geometry. TEER, as a leaflet-based repair strategy, does not directly correct annular enlargement. Although this approach may restore leaflet coaptation acutely, its long-term durability in a predominantly annular disease remains to be fully elucidated. Emerging registry data suggest that TEER is safe and associated with symptomatic improvement in patients with atrial phenotypes, particularly in elderly or high-risk populations, by enhancing leaflet coaptation [7,39,40,41,42].

However, the predominantly annular nature of A-SMR raises concerns regarding long-term durability. Progressive annular dilatation and ongoing atrial remodeling may continue despite successful edge-to-edge repair, predisposing to residual or recurrent MR over time [4,43]. In a registry-based analysis of patients undergoing TEER for A-SMR, extreme annular enlargement and markers of advanced atrial myopathy were associated with higher rates of residual or recurrent MR during follow-up [43].

By contrast, surgical annuloplasty directly targets annular remodeling and has shown favorable durability in atrial-dominant phenotypes [8]. Nevertheless, comparative long-term data between TEER and surgery specifically in pure A-SMR remain limited. These considerations underscore the importance of mechanism-oriented patient selection and highlight the need for prospective studies specifically addressing TEER durability in atrial-dominant disease. Patients with moderate annular dilatation, preserved leaflet tissue, and less advanced atrial remodeling appear more likely to benefit, whereas those with advanced atrial disease and severe TR may experience limited or transient improvement [4].

To provide a structured comparison between available treatment strategies in A-SMR, we summarize current comparative evidence in Table 1. Although no randomized controlled trials have directly compared surgical annuloplasty and TEER in pure A-SMR, a recent meta-analysis of 32 observational studies including 1923 patients provides the most comprehensive comparative assessment currently available [44]. In this analysis, TEER patients were on average approximately 10 years older, had a higher prevalence of New York Heart Association functional classification (NYHA) class III–IV symptoms, and a greater surgical risk profile. Early outcomes—including 30-day mortality, stroke, and acute kidney injury—were comparable between strategies, with no significant differences in pooled early event rates. At a weighted mean follow-up of 3.2 years (longer in surgical cohorts), surgery was associated with significantly lower incidence rates of late (i.e., beyond 30 days after the index procedure) severe MR (2.53 vs. 6.66 events per 100 person-years; *p* for interaction = 0.03), late all-cause mortality (3.00 vs. 8.84 per 100 person-years; *p* = 0.02), HF hospitalization (4.44 vs. 17.03 per 100 person-years; *p* < 0.01), and persistent NYHA III–IV status (2.98 vs. 22.47 per 100 person-years; *p* < 0.01). However, substantial between-study heterogeneity was observed for several long-term endpoints (I^2^ up to ~70–90%), and no randomized comparisons were available. Importantly, TEER cohorts were generally older, more symptomatic, and had shorter follow-up durations, introducing significant selection bias and limiting causal inference. Therefore, while surgery appeared associated with more durable long-term outcomes in this pooled analysis, these findings should be interpreted cautiously and considered hypothesis-generating rather than definitive comparative evidence.

## 7. Surgical Implications in Atrial Secondary Mitral Regurgitation

Surgical treatment represents a cornerstone in the management of A-SMR, as it directly addresses the dominant anatomical mechanism underlying the disease. In contrast to V-SMR, where surgical outcomes are strongly influenced by left ventricular geometry and function, A-SMR is primarily an annular disorder occurring in the setting of preserved ventricular performance. This fundamental distinction has important implications for patient selection, timing of intervention, and choice of surgical technique [8,15].

### 7.1. Indications and Timing for Surgery

Surgical intervention should be considered in patients with severe A-SMR who remain symptomatic despite optimized medical therapy and appropriate rhythm or rate control strategies, in accordance with 2025 ESC guidelines recommendations (Class IIa Level B Recommendation) [5]. Timing of intervention is particularly relevant, as surgery performed before the development of advanced pulmonary hypertension or right ventricular dysfunction is more likely to result in durable mitral valve repair and favorable clinical outcomes [45].

### 7.2. Surgical Techniques (Table 2)

**Figure 3:**
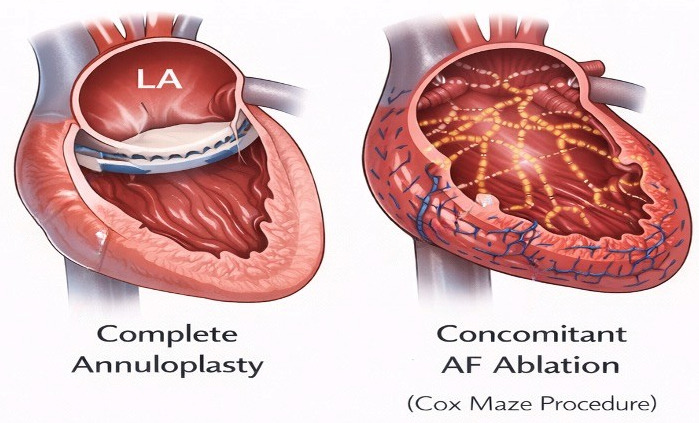
Mitral valve annuloplasty and AF Ablation. Abbreviations: AF: Atrial Fibrillation; LA: Left Atrium.

Mitral valve repair based on annuloplasty remains the preferred surgical strategy for A-SMR, as it directly targets the primary mechanism of annular dilatation [46] in the presence of structurally normal leaflets and an intact subvalvular apparatus (Figure 3). Restoration of annular size and geometry improves leaflet coaptation and mitral competence without the need for ventricular or papillary muscle–directed interventions [8].

Early clinical evidence supporting this approach was provided by Vohra et al. [47], who reported favorable mid-term outcomes in 20 patients undergoing systematic mitral ring annuloplasty for severe MR secondary to lone AF. There was no in-hospital mortality, and post-repair TEE showed no residual MR in 90%, with only mild MR in 10%. At discharge, significant reverse remodeling was observed, with reductions in Left Ventricular End-Diastolic Diameter (LVEDD) (5.6 ± 0.7 to 4.8 ± 0.7 cm; *p* < 0.005), Left Ventricular End-Systolic Diameter (LVESD) (4.0 ± 0.7 to 3.2 ± 0.8 cm; *p* < 0.005), and LA diameter (6.1 ± 1.6 to 5.2 ± 1.0 cm; *p* = 0.03), together with a decrease in systolic pulmonary artery pressure (54.1 ± 12.2 to 40.4 ± 15.5 mmHg; *p* = 0.02). Functional status improved markedly, with 85% of patients in NYHA class I/II at latest follow-up (*p* < 0.0001 vs. preoperative) and no deaths, thromboembolic events, endocarditis, reoperation, or recurrent severe MR over a mean follow-up of 18.0 ± 12.5 months.

Subsequent studies have consistently confirmed annuloplasty as the foundation of surgical repair in this setting.

In a single-center retrospective study of 162 patients with A-SMR undergoing mitral valve repair, Shin et al. reported durable mid- to long-term outcomes. Repair was predominantly performed with complete ring annuloplasty tailored to commissural dimensions. Over a median follow-up of 6.1 years, 5- and 10-year survival rates were 86% and 73%, respectively, while recurrent moderate-or-greater MR occurred in 13.5% of patients (freedom from recurrence 89% at 5 years and 80% at 10 years). On multivariable analysis, age (HR 1.095 per year; *p* < 0.001), AF (HR 2.30; *p* = 0.048), and chronic kidney disease (HR 4.17; *p* = 0.048) independently predicted mortality, whereas LAVi predicted MR recurrence (HR 1.014 per mL/m^2^; *p* = 0.028) [48].

A comprehensive scoping review of the surgical literature [49] further demonstrated that, despite substantial heterogeneity in diagnostic criteria and patient selection, mitral valve repair with ring annuloplasty represents the predominant surgical strategy across published A-SMR series. Across 12 studies including 494 patients, isolated mitral annuloplasty was performed in 96.2% of cases. However, the use of adjunctive leaflet, atrial, and rhythm-control procedures remained highly variable, with concomitant neochordoplasty reported in 13.3–55% of patients, posterior leaflet augmentation in 24.4% in selected series, and the Cox–Maze AF ablation procedure performed in only a median of 40.9% of patients (IQR 21.3–63.4%). Notably, a median of 51.5% (IQR 35.0–78.7%) of patients did not undergo any surgical ablation for AF, underscoring the absence of standardized treatment algorithms.

Disease progression plays a key role in determining the adequacy of isolated annuloplasty. As highlighted by Aranda-Domene and colleagues, A-SMR may evolve from an early stage dominated by annular dilatation and central regurgitation to more advanced stages characterized by atriogenic posterior leaflet tethering, anterior leaflet pseudoprolapse, and eccentric regurgitant jets. In such cases, annuloplasty alone may be insufficient, prompting consideration of more comprehensive surgical strategies [50].

Several authors have therefore advocated adjunctive techniques tailored to advanced atrial remodeling, including leaflet augmentation and atrial plication [50,51,52,53]. In the presence of anterior leaflet pseudo-prolapse related to malcoaptation, implantation of artificial chordae has been proposed to restore leaflet alignment; however, the supporting evidence remains limited to small surgical experiences. For example, in a reported mid-term series of 40 A-SMR repairs, artificial chordae replacement for anterior leaflet pseudo-prolapse was used in 19 patients, and recurrent moderate-to-severe MR occurred in 2 patients during follow-up. In more advanced phenotypes with a giant LA, marked annular enlargement, and a relatively small posterior leaflet, posterior leaflet augmentation using an autologous pericardial patch has been advocated to increase coaptation area; nevertheless, concerns persist regarding long-term durability because patch shrinkage, thickening, and calcification have been described, and in one institutional experience patch augmentation was performed in 16 advanced cases with 2 valve-related reoperations reported [51,52].

Matsumori et al. [53] retrospectively evaluated the impact of adjunctive left atrial plication (LAP) in 22 patients with A-SMR, permanent AF (≥3 years), left atrial enlargement, and preserved LVEF. Patients were divided into LAP+ (*n* = 9) and LAP− (*n* = 13). Mitral procedures included ring annuloplasty in 16 patients and valve replacement in 6, with adjunctive leaflet procedures performed more frequently in the LAP−group. The primary morphologic endpoint, change in mitral valve angle assessed by CT, was significantly greater in the LAP+ group (−16.6 ± 8.1° vs. −1.2 ± 6.9°, *p* < 0.01), accompanied by larger reductions in left atrial dimension (−18.4 ± 7.0 mm vs. −6.9 ± 14.6 mm, *p* = 0.02) and cardiothoracic ratio (−11.9 ± 4.7% vs. −2.7 ± 6.0%, *p* < 0.01). There was no in-hospital mortality. During a mean clinical follow-up of 3.6 ± 3.0 years, no HF rehospitalizations, cardiac deaths, or MR recurrence were observed. These findings suggest that LAP may provide additional geometric remodeling and may enhance repair durability in selected patients with advanced atrial enlargement.

Takahashi et al. reported sustained reduction of MR and marked symptomatic improvement following mitral valve repair in 10 patients with chronic AF and atrial functional MR, despite the absence of durable rhythm restoration. All patients underwent ring annuloplasty (median ring size 26 mm) with concomitant tricuspid annuloplasty and left atrial appendage (LAA) closure; 30-day mortality was 0%, and no heart-failure readmissions occurred during follow-up (10–52 months). Left atrial remodeling was documented by a significant reduction in LAVi from 72 ± 26 to 48 ± 17 mL/m^2^ at latest follow-up (*p* = 0.03), while functional status improved from NYHA 3.0 ± 0.7 to 1.2 ± 0.4 (*p* < 0.0001). Notably, although only two patients transiently regained sinus rhythm after a Maze procedure and all had persistent AF at latest follow-up, MR remained mild or improved in all patients, supporting the concept that correcting annular pathology can provide clinical benefit even without rhythm restoration [54].

Nevertheless, outcomes appear less favorable in patients with excessive atriogenic or combined atriogenic–ventriculogenic leaflet tethering. In a small retrospective series of patients with A-SMR and preserved LVEF, mitral annuloplasty resulted in significant symptomatic and echocardiographic improvement, although recurrent MR occurred in 20% of cases [55]. Preoperative LV dilatation and increased leaflet tethering were associated with recurrence, suggesting that annuloplasty alone may be insufficient in more advanced phenotypes. Accordingly, the authors proposed a mechanism-oriented surgical strategy, whereby isolated annuloplasty may be adequate in pure annular dilatation, whereas undersized annuloplasty or adjunctive techniques—and even valve replacement in severe tethering—should be considered when atriogenic tethering is present.

Given the frequent coexistence of atrial functional TR, several studies have emphasized the need for an integrated, dual-valve approach [47,52,54,56]. Combined mitral and tricuspid annuloplasty has been shown to result in sustained reduction of both regurgitations and improved functional status, while preoperative LAVi has emerged as an important predictor of late CV events, reinforcing the prognostic role of advanced atrial remodeling [56]. Notably, this concept has been acknowledged in the most recent 2025 ESC guidelines on valvular heart disease, which recommend concomitant tricuspid valve (TV) intervention in patients with severe TR undergoing left-sided valve surgery and suggest consideration of tricuspid repair even in moderate or mild regurgitation in the presence of annular dilatation, with the aim of preventing disease progression and right ventricular remodeling [5].

Parallel to these mechanistic advances, surgical strategies for A-SMR have evolved alongside the widespread adoption of minimally invasive mitral valve surgery [57]. Contemporary minimally invasive platforms allow the full spectrum of mitral repair and concomitant atrial procedures to be performed with outcomes comparable to conventional sternotomy, while offering a less invasive surgical framework [57].

Balogh et al. [58] investigated the long-term impact of isolated minimally invasive endoscopic mitral valve repair in patients with HFpEF and A-SMR. In this single-center retrospective study, 131 patients undergoing MV repair were compared with 139 patients treated with standard care using propensity-score–based methods and inverse probability weighting. Over a median follow-up of 5.0 years, MV repair was associated with significantly lower all-cause mortality (1-year: 1% vs. 9%; 5-year: 12% vs. 40%; both *p* ≤ 0.002) and fewer HFpEF readmissions (1-year: 4% vs. 17%; 5-year: 10% vs. 34%; *p* ≤ 0.006) compared with standard care. In adjusted Cox analysis, MV repair emerged as the strongest independent predictor of improved outcomes, with hazard ratios of 0.16 (95% CI 0.07–0.34; *p* < 0.001) for all-cause mortality and 0.21 (95% CI 0.09–0.51; *p* < 0.001) for HFpEF readmissions. These findings suggest that isolated endoscopic mitral repair may provide substantial long-term clinical benefit in selected HFpEF patients with A-SMR.

**Table 2:** Summary of contemporary surgical approaches for atrial secondary mitral regurgitation.

Study	Population	Surgical Strategy	Key Findings	Statistical Outcomes
**Vohra et al.** [47]	*n* = 20 severe A-SMR (lone AF)	Ring annuloplasty ± AF surgery ± TV repair	Significant reverse remodeling and symptomatic improvement	No in-hospital mortality; MR none/trivial 90%, mild 10%; LVEDD decreased (*p* < 0.005), LVESD decreased (*p* < 0.005), LA diameter decreased (*p* = 0.03), sPAP decreased (*p* = 0.02); 85% NYHA I/II at follow-up
**Shin et al.** [48]	*n* = 162 A-SMR	Predominantly complete ring annuloplasty	Durable long-term outcomes	5- and 10-years survival 86% and 73%; MR recurrence 13.5% (freedom 89% at 5 years, 80% at 10 years); independent predictors of mortality: age HR 1.095 (*p* < 0.001), AF HR 2.30 (*p* = 0.048), CKD HR 4.17 (*p* = 0.048); MR recurrence predicted by LAVi HR 1.014 (*p* = 0.028)
**Amabile et al. (Scoping review)** [49]	12 surgical studies, *n*= 494 A-SMR patients	Annuloplasty-based MV repair	Marked heterogeneity in A-SMR definitions and adjunctive procedures; annuloplasty is the predominant strategy	Annuloplasty in 96.2%; AF used to define A-SMR in 75% of studies; AF > 1 year required in 41.2%; LVEF thresholds 45–55%; Cox-Maze range 17.8–79.5%, PVI 0–66.7%, LAA ligation 0–100%; neochordoplasty 13.3–55%, posterior leaflet extension 24.4%.
**Nappi (Review)** [51]	Comprehensive A-SMR review	Annuloplasty-based integrated management	Confirms A-SMR as atrial–annular disease requiring tailored strategy	No original surgical statistics; reports cited data (e.g., MR higher with AF recurrence after ablation: 82% vs. 24%, *p* = 0.005)
**Shibata et al. (Review)** [52]	Narrative review (A-SMR/A-STR)	Mechanism-oriented surgical framework	Advanced A-SMR may require adjunctive techniques beyond annuloplasty	No original statistical analysis; cites small series (e.g., neochords in 19/40; patch augmentation in 16 cases with 2 reoperations)
**Matsumori et al.** [53]	*n* = 22 A-SMR (permanent AF ≥ 3 years), LAP+ (*n* = 9) vs. LAP− (*n* = 13)	MV surgery ± LAP	LAP associated with greater geometric reverse remodeling; no MR recurrence reported	ΔMV angle −16.6 ± 8.1° vs. −1.2 ± 6.9° (*p* < 0.01); ΔLA dimension −18.4 ± 7.0 vs. −6.9 ± 14.6 mm (*p* = 0.02); ΔCTR −11.9 ± 4.7% vs. −2.7 ± 6.0% (*p* < 0.01); no in-hospital deaths; no MR recurrence during follow-up (mean 3.58 ± 3.01 years)
**Takahashi et al.** [54]	*n* = 10 A-SMR with chronic AF	Ring annuloplasty + TV repair + LAA closure	Sustained MR reduction and symptomatic improvement despite persistent AF	30-day mortality 0%; no HF readmissions; MR mild/improved in all; LAVi ↓ 72 ± 26→48 ± 17 mL/m^2^ (*p* = 0.03); NYHA ↓ 3.0 ± 0.7→1.2 ± 0.4 (*p* < 0.0001)
**Sakaguchi et al.** [55]	*n* = 20 A-SMR with chronic AF	Mitral ring annuloplasty	Symptomatic and echocardiographic improvement, but recurrence in advanced phenotypes	Recurrent ≥ moderate MR 20% (2 reoperations); NYHA ↓ 2.3 ± 0.6→1.3 ± 0.6 (*p* < 0.0001); LAVi ↓ 94 ± 59→58 ± 30 mL/m^2^ (*p* = 0.0014); TR gradient ↓ 34 ± 11→23 ± 5 mmHg (*p* = 0.0004); recurrence associated with larger LV dimensions (*p* = 0.019) and greater leaflet tethering
**Takahashi et al.** [56]	*n* = 45 permanent AF, preserved LVEF, ≥moderate A-SMR + ≥mild A-STR	Combined mitral + tricuspid annuloplasty (100%); adjunct PML extension 24%, chordal loops 33%, LAA closure 67%, Maze 18%	Significant and sustained reduction of MR/TR and improved functional status; LAVi predicts late cardiovascular events	MR score 2.6 ± 0.6→0.9 ± 0.5 (*p* < 0.0001) and TR score 2.0 ± 0.7→0.8 ± 0.5 (*p* < 0.0001); NYHA 2.8 ± 0.7→1.5 ± 0.7 (*p* < 0.0001); event-free rates 93%, 87%, 52% at 1/3/5 years; LAVi independent predictor of CV events (HR 1.01, 95% CI 1.00–1.02; *p* = 0.0028)
**Balogh et al.** [58]	HFpEF (LVEF ≥50%, H2FPEF ≥5) + A-SMR: MV Repair *n* = 131 vs. StanCare *n* = 139 (IPTW)	Minimally invasive endoscopic MV repair (undersized semi-rigid ring) ± TV annuloplasty/maze	MV repair associated with lower long-term mortality and HFpEF readmissions vs. standard care	30-day mortality 1%; redo MV surgery 1.4%; 1-year and 5-years mortality 1% vs. 9% (*p* = 0.002) and 12% vs. 40% (*p* < 0.001); HF readmissions 1-year and 5-years 4% vs. 17% (*p* = 0.006) and 10% vs. 34% (*p* < 0.002); adjusted HR for mortality 0.16 (95% CI 0.07–0.34; *p* < 0.001) and for HF readmission 0.21 (95% CI 0.09–0.51; *p* < 0.001)

Abbreviations: A-SMR, atrial secondary mitral regurgitation; A-STR, atrial secondary tricuspid regurgitation; AF, atrial fibrillation; CKD, chronic kidney disease; CTR, cardiothoracic ratio; CV, cardiovascular; H2FPEF, H2FPEF score (clinical score for HFpEF probability); HF, heart failure; HFpEF, heart failure with preserved ejection fraction; HR, hazard ratio; IPTW, inverse probability of treatment weighting; LA, left atrium; LAA, left atrial appendage; LAP, left atrial plication; LAVi, left atrial volume index; LV, left ventricle; LVEDD, left ventricular end-diastolic diameter; LVEF, left ventricular ejection fraction; LVESD, left ventricular end-systolic diameter; MR, mitral regurgitation; NYHA, New York Heart Association functional class; PML, posterior mitral leaflet; PVI, pulmonary vein isolation; sPAP, systolic pulmonary artery pressure; TR, tricuspid regurgitation; TV, tricuspid valve.

#### Comparative Outcomes and Predictors of Durability

Comparative surgical studies have further clarified the distinct prognostic profile of A-SMR. Compared with V-SMR, patients with A-SMR exhibit superior long-term survival and lower rates of reoperation following mitral valve surgery, despite similar early operative risk [15]. Importantly, etiological classification has emerged as a key determinant of outcome, supporting tailored surgical decision-making [59].

The choice between partial band and complete ring annuloplasty in A-SMR remains a relevant technical consideration. Although several surgical series have identified partial posterior bands as an independent predictor of recurrent MR [60,61], their continued use reflects specific technical and physiological considerations rather than simple preference. Partial bands primarily address posterior annular dilatation while preserving anterior annular and saddle-shaped dynamics. In patients with moderate annular enlargement and preserved leaflet tissue, this approach may reduce regurgitation while limiting the risk of excessive annular restriction [62]. However, in A-SMR—where annular dilatation is typically circumferential and associated with progressive atrial remodeling—a complete rigid or semi-rigid ring provides more uniform annular stabilization and may better prevent late redilatation. Ring sizing represents a critical determinant of both durability and postoperative valve hemodynamics [63]. Undersizing enhances leaflet coaptation and reduces recurrent MR but increases the risk of functional mitral stenosis, particularly in patients with small baseline annular dimensions or reduced leaflet reserve [63].

In A-SMR, where leaflet tethering is usually limited and coaptation failure is predominantly annular-driven, extreme downsizing may be unnecessary. A balanced strategy consists of selecting a complete ring sized according to the anterior leaflet height or intertrigonal distance, avoiding aggressive downsizing beyond one size below the measured annular dimension, and verifying intraoperative transmitral gradients under physiologic flow conditions [64]. Maintenance of an adequate mitral valve area and mean gradient (<5 mmHg at normal heart rates) is essential, especially in elderly patients or those with concomitant AF and reduced diastolic filling time [65]. In patients with borderline annular dimensions or high risk of functional mitral stenosis, moderate undersizing with a complete ring may provide optimal annular remodeling while preserving valve area [64]. Overall, given the predominantly annular pathophysiology of A-SMR, circumferential stabilization with an appropriately sized complete ring appears mechanistically advantageous, whereas partial bands may be reserved for selected cases with limited posterior dilatation and preserved annular geometry [1,63].

Within the A-SMR population, long-term follow-up studies extending up to 10 years have provided valuable insights into repair durability [60]. Kawamoto et al. conducted a retrospective analysis of 50 consecutive patients with A-SMR undergoing mitral valve surgery to identify predictors of recurrent MR and mid-term outcomes. Mitral repair with annuloplasty was performed in 42 patients and valve replacement in 8. Over a mean follow-up of 4.6 ± 4.4 years, freedom from cardiac death was 88% at 5 years and 78.6% at 10 years, with better survival observed after repair compared with replacement (log-rank *p* = 0.04). Recurrent moderate-or-greater MR after repair occurred in 16.8% at both 5 and 10 years, mainly due to annuloplasty dehiscence or inadequate leaflet coaptation. On multivariable analysis, the use of partial band annuloplasty emerged as the only independent predictor of recurrence (HR 10.1; *p* = 0.049), supporting preferential use of complete annuloplasty rings to improve repair durability in A-SMR [60].

The use of partial band annuloplasty has also been investigated by Kwon et al. [61]. In a large single-center retrospective study of 548 patients undergoing annuloplasty for functional MR, Kwon et al. compared outcomes between partial band and complete ring annuloplasty. Among 479 patients with echocardiographic follow-up, recurrent moderate-or-greater MR occurred significantly more often after partial band repair than after complete ring annuloplasty (21% vs. 10%, *p* = 0.001), a finding that persisted after propensity matching (17% vs. 7%, *p* = 0.049). On multivariable analysis, use of a partial band was an independent predictor of recurrence (OR ≈ 2.0, *p* = 0.012), along with preoperative severe MR, although overall survival did not differ between groups. The authors concluded that complete rings provide more durable annular stabilization and reduce MR recurrence compared with partial bands in functional MR (Table 2).

Rigid or semi-rigid complete annuloplasty rings are therefore generally favored to prevent recurrent annular dilatation, with careful sizing required to balance effective annular reduction against the risk of functional mitral stenosis [15]. In contrast to V-SMR, subvalvular repair techniques aimed at modifying papillary muscle position are typically unnecessary in A-SMR, reflecting the limited contribution of ventricular distortion to disease pathophysiology [15].

### 7.3. Concomitant Atrial Fibrillation Surgery and Left Atrial Appendage Closure

Given the central role of AF in the development and progression of A-SMR, concomitant surgical ablation should be strongly considered at the time of mitral valve repair. Surgical ablation strategies (Figure 3) aim to restore sinus rhythm, reduce AF burden, and promote atrial reverse remodeling. This integrated approach addresses both the cause and the consequence of atrial-driven MR [66,67].

Chen et al. [67] reported one of the largest surgical series evaluating mitral valve repair for A-SMR with a specific focus on the impact of concomitant surgical ablation for AF. In a cohort of 82 patients with persistent AF, preserved left ventricular systolic function, and moderate-to-severe A-SMR, all patients underwent mitral valve repair with annuloplasty, and 63.4% received concomitant Cox–Maze IV ablation. Cox–Maze IV ablation is a standardized surgical ablation technique that creates a predefined pattern of linear atrial lesions using bipolar radiofrequency and/or cryoablation, reproducing the lesion set of the original cut-and-sew Cox–Maze III procedure while reducing operative complexity and morbidity. Surgical treatment was associated with significant reverse remodeling, including reductions in left atrial diameter, LV dimensions (all *p* < 0.001), and pulmonary artery pressure (*p* = 0.006), with no operative mortality and 96.1% of patients improving to NYHA class I/II. During a mean follow-up of 26.1 ± 27.6 months, freedom from recurrent MR at 3 years was significantly higher in the surgical ablation group compared with the non-ablation group (93.8% vs. 44.2%; log-rank *p* = 0.035). Propensity score–adjusted and IPTW Cox analyses confirmed a significant interaction between surgical ablation and left atrial size, with the benefit of ablation on MR recurrence confined to patients with smaller left atrial diameter (≤60 mm), whereas no significant advantage was observed in those with advanced atrial enlargement.

Available evidence suggests that restoration of sinus rhythm may improve atrial function and annular dynamics, potentially enhancing the durability of mitral valve repair [6].

Ye et al. [68] reported a large prospective cohort study evaluating outcomes of mitral valve repair combined with the Cox–Maze procedure in patients with A-SMR and HF with recovered LVEF. Among 312 consecutively enrolled patients, 247 underwent mitral valve repair plus surgical ablation after guideline-directed medical therapy, including 132 with full LVEF recovery (≥50%) and 115 with partial recovery (40–50%). Operative mortality was low and comparable between groups (0.8% vs. 0.9%), and overall 5-year survival reached 95% in the total cohort. Importantly, although IPW-adjusted overall survival was similar between groups (HR 2.18, 95% CI 0.46–10.38; *p* = 0.33), patients with LVEF ≥ 50% demonstrated significantly better long-term freedom from recurrent MR (HR 2.44, 95% CI 1.28–4.63; *p* = 0.0065) and AF recurrence (HR 1.85, 95% CI 1.06–3.21; *p* = 0.030) compared with those with partial recovery. After inverse probability weighting, HF rehospitalization rates were not significantly different (HR 1.36, 95% CI 0.91–2.03; *p* = 0.13). The authors concluded that mitral valve repair combined with Cox–Maze is safe and effective in patients with A-SMR and recovered systolic function, with more durable rhythm and valve outcomes observed in those achieving full ventricular recovery.

Song et al. [69] compared surgical outcomes of mitral valve surgery in patients with A-SMR and degenerative mitral regurgitation (DMR) in a large multicenter cohort of 642 patients, including 82 propensity score–matched patients with A-SMR. Mitral valve repair was the predominant strategy in both groups (89.0% in A-SMR vs. 92.7% in DMR; *p* = 0.333) and was associated with a similarly low rate of recurrent MR, with 5-year freedom from moderate-or-greater MR of 93.0% in A-SMR and 89.8% in DMR (log-rank *p* = 0.699). Despite comparable durability of mitral repair, patients with A-SMR experienced higher rates of HF–related readmission and cardiac death at 5 years (freedom from the composite endpoint 88.6% vs. 96.3%; log-rank *p* = 0.045; freedom from cardiac death 90.0% vs. 100%; *p* = 0.002), reflecting the impact of advanced atrial remodeling. Importantly, following concomitant Maze procedures, A-SMR patients showed a significantly higher incidence of junctional rhythm (49.1% vs. 3.3%; *p* < 0.001) and permanent pacemaker implantation during follow-up (5-year freedom 86.0% vs. 98.3%; log-rank *p* = 0.007), highlighting a distinctive rhythm-related risk profile in this population. Overall, the study supports mitral valve repair as an effective surgical option for A-SMR while emphasizing the need for careful rhythm management and postoperative surveillance.

LAA closure at the time of mitral valve intervention is often considered to reduce the long-term risk of stroke in patients with permanent AF, particularly in those with contraindications to long-term anticoagulation [35]. While dedicated randomized data in A-SMR populations are limited, expert consensus and recent evidence support the integration of LAA closure into the surgical strategy for selected patients with persistent or permanent AF, as part of a comprehensive approach to atrial remodeling and thromboembolic risk reduction [8,46,70].

## 8. Clinical–Surgical Decision-Making: Algorithm Proposal

Clinical decision-making in A-SMR should be guided by a mechanism-oriented approach that integrates clinical presentation, imaging findings, and procedural risk. Given the fundamental differences between atrial-driven and ventricular functional MR, accurate identification of A-SMR is the cornerstone of appropriate management and therapeutic selection [10,13].

Patients presenting with functional MR and HFpEF should undergo comprehensive echocardiographic evaluation to confirm the atrial mechanism. Key features supporting a diagnosis of A-SMR include significant left atrial enlargement, mitral annular dilatation, minimal leaflet tethering, and the absence of primary mitral valve disease [22,23]. Recognition of these characteristics is essential to avoid misclassification and inappropriate application of treatment strategies designed for V-SMR.

In asymptomatic or mildly symptomatic patients with early-stage A-SMR, initial management should focus on optimization of medical therapy and treatment of associated conditions, including HFpEF and AF [5,6]. Rhythm control strategies may be particularly beneficial in patients with limited atrial remodeling, as restoration of sinus rhythm can promote atrial reverse remodeling and reduce MR severity [6].

Patients with persistent symptoms despite optimized non-surgical management require further stratification based on atrial size, annular dimensions, pulmonary pressures, and right ventricular function. Progressive annular dilatation, persistent AF, and early pulmonary hypertension identify patients at higher risk of disease progression and adverse outcomes if intervention is delayed [45].

In patients who are suitable surgical candidates, mitral valve repair based on annuloplasty should be considered the preferred treatment strategy, particularly when intervention is performed before the development of advanced pulmonary hypertension or right ventricular dysfunction [45]. Concomitant surgical ablation of AF should be strongly encouraged, as it addresses the underlying atrial pathology and may enhance the durability of mitral repair [6,8].

TEER may be considered in selected patients who are deemed at prohibitive surgical risk or are not candidates for surgery. In these cases, careful patient selection is critical, as the annular-driven nature of A-SMR may limit long-term durability, particularly in the presence of extreme annular enlargement or advanced atrial myopathy [7,43].

In addition to pure atrial phenotypes, clinicians frequently encounter patients with overlapping atrial and ventricular mechanisms, which require tailored consideration.

### Management of the Dual Atrial–Ventricular Phenotype

In contemporary cohorts, a substantial proportion of patients present with mixed atrial and ventricular mechanisms of functional MR (almost 50% of the patients with functional MR) [3]. In these “dual phenotype” cases, a mechanism-oriented strategy is essential, as isolated annuloplasty may be insufficient when a relevant ventricular component is present. Quantitative imaging parameters can assist in defining the dominant mechanism.

Three-dimensional echocardiographic analysis demonstrates that atrial and ventricular functional MR share significant annular dilatation and reduced annular contraction; however, they differ fundamentally in leaflet geometry. In atrial MR, left ventricular size and systolic function are preserved, and leaflet tethering is minimal (tenting height ≈ 3–4 mm), indicating preserved subvalvular alignment. Conversely, V-SMR is characterized by left ventricular dilatation, reduced LVEF, and marked leaflet tethering (tenting height ≈ 8 mm), reflecting papillary muscle displacement. Despite similar reductions in coaptation index in both phenotypes, the underlying mechanism of coaptation failure is annular-driven in atrial MR and tethering-driven in ventricular MR [71].

In patients with predominant atrial disease, complete annuloplasty alone is generally sufficient. However, when ventricular remodeling and leaflet tethering are substantial, isolated annuloplasty may be associated with a higher risk of recurrence. In this setting, treatment decisions should be individualized by the Heart Team, balancing the role of optimized guideline-directed medical therapy, cardiac resynchronization when indicated, and TEER in appropriately selected patients, in accordance with contemporary ESC/EACTS recommendations [5,72].

Ultimately, management decisions in A-SMR should be individualized and guided by a multidisciplinary Heart Team approach. Integration of clinical status, imaging phenotype, procedural risk, and patient preferences is essential to align treatment strategy with disease mechanism and stage, thereby optimizing clinical outcomes [5].

## 9. Future Directions

Despite increasing recognition of A-SMR as a distinct clinical and mechanistic entity, several important knowledge gaps remain, particularly with regard to optimal timing of intervention, patient selection, and surgical strategy. Addressing these gaps is essential to refine management algorithms and improve long-term outcomes. From a surgical standpoint, future research should prioritize prospective, phenotype-specific studies that clearly distinguish A-SMR from V-SMR and dual-mechanism functional MR [3,13,15]. The heterogeneity of current surgical series, often including mixed phenotypes, limits the ability to draw firm conclusions regarding durability and outcomes of mitral valve repair in pure A-SMR. Standardized diagnostic and imaging criteria integrating annular geometry, leaflet–annulus mismatch, atrial size, and atrial fibrosis burden are needed to improve surgical candidate selection and procedural planning [22,23,24,28,30]. The timing of surgical intervention represents a particularly relevant area for future investigation. In observational surgical series, earlier intervention, before advanced pulmonary hypertension or RV dysfunction, was associated with superior durability and clinical outcomes [2,45]. However, prospective data defining optimal intervention thresholds are lacking. Future studies comparing early surgical referral versus delayed intervention in patients with progressive A-SMR may clarify whether timely annuloplasty-based repair can interrupt the self-perpetuating cycle of atrial remodeling and regurgitation progression. Further refinement of surgical techniques tailored to disease stage is also warranted. While complete rigid or semi-rigid annuloplasty is widely accepted as the cornerstone of repair in A-SMR [46,60], the role of adjunctive procedures—such as leaflet augmentation, atrial plication, or other atrial remodeling strategies—requires systematic evaluation. Identifying which patients benefit from isolated annuloplasty versus more complex repair strategies remains an unmet clinical need, particularly in advanced stages characterized by atriogenic leaflet tethering [50,51,52,54]. The integration of concomitant AF surgery represents another key area for future research. Available evidence suggests that surgical ablation may reduce AF burden, promote atrial reverse remodeling, and improve the durability of mitral valve repair in selected patients [6,67,68]. However, the benefit of ablation appears attenuated in patients with advanced atrial enlargement and extensive atrial fibrosis [30,32,67]. Future studies should aim to define atrial size, fibrosis extent, and AF duration thresholds beyond which the incremental benefit of surgical ablation becomes limited. Finally, comparative studies specifically focused on surgical repair versus transcatheter therapies in A-SMR populations are needed. While TEER represents a valuable option for high-risk or inoperable patients [5,7,36,39,40,41], concerns remain regarding long-term durability in a predominantly annular disease characterized by progressive atrial remodeling [4,43]. Direct comparisons incorporating anatomical, functional, and patient-reported outcomes will be essential to better define the respective roles of surgical and transcatheter interventions within a mechanism-oriented treatment framework.

## 10. Conclusions

A-SMR is a distinct and increasingly recognized form of functional MR driven primarily by left atrial remodeling and mitral annular dilatation in the setting of preserved ventricular geometry and systolic function [1,3,10]. Its recognition challenges the traditional ventricular-centered paradigm of functional mitral valve disease and has major implications for diagnosis, risk stratification, and therapeutic decision-making. From a cardio-surgical perspective, A-SMR is fundamentally an annular disease, making mitral valve repair based on complete annuloplasty the most pathophysiologically sound and definitive therapeutic option in suitable candidates [8,15,46]. In selected advanced phenotypes, adjunctive surgical techniques may be required to optimize repair durability, including leaflet augmentation, artificial chordae implantation, or LAP, to address atriogenic leaflet tethering and extreme annular–atrial remodeling [50,51,52,53,54]. Moreover, TEER may represent a reasonable alternative in high-risk or inoperable patients, although it does not directly correct the underlying annular pathology [39,40,41,42,43]. Compared with V-SMR, patients with A-SMR generally demonstrate superior long-term survival and lower rates of reoperation following surgical repair, underscoring the importance of etiological classification in surgical decision-making [15,59]. The frequent coexistence of AF and atrial functional TR further supports a comprehensive and integrated surgical strategy, combining mitral annuloplasty with concomitant AF ablation, TV repair, and, when appropriate, LAA closure [47,52,53,55,67,68,69,70]. This approach targets both the cause and consequences of atrial remodeling and may enhance long-term valve competence and clinical stability. Nevertheless, surgical outcomes are strongly influenced by disease stage at the time of intervention. Established pulmonary hypertension is consistently associated with higher rates of recurrent MR and less favorable long-term outcomes [45]. These observations highlight the critical importance of timely referral and intervention before irreversible atrial and right-sided remodeling develops. In conclusion, A-SMR should be managed through a mechanism-oriented, multidisciplinary Heart Team approach, with cardiac surgery playing a central role in eligible patients. Continued refinement of diagnostic criteria, surgical techniques, and timing strategies—supported by dedicated prospective studies—will be essential to optimize outcomes and fully define the role of surgical therapy in this increasingly prevalent and clinically relevant disease phenotype.

## Figures and Tables

**Table 1:** Comparative outcomes of surgical annuloplasty versus TEER in A-SMR [44].

Outcome	Surgical Repair	TEER	*p* for Interaction
**Study characteristics**			
Population (studies/patients)	20 studies/1166 pts	12 studies/757 pts	—
Mean age (years)	~69.5	~79.4	—
Pre-procedural NYHA III–IV (%)	~41%	~82%	—
Mean follow-up (years)	4.48	1.41	—
**Clinical outcomes**			
Late severe MR (events/100 PY)	2.53 (1.66–3.84)	6.66 (3.09–14.32)	0.03
Late all-cause mortality (events/100 PY)	3.00 (1.57–5.72)	8.84 (4.47–17.47)	0.02
Late HF hospitalization (events/100 PY)	4.44 (2.16–9.14)	17.03 (13.17–22.03)	<0.01
Late NYHA III–IV (events/100 PY)	2.98	22.47	<0.01
Early all-cause mortality	Similar	Similar	NS
Early stroke	Similar	Similar	NS
Early AKI	Similar	Similar	NS
Late reoperation	Similar	Similar	NS

Abbreviations: A-SMR, Atrial Secondary Mitral Regurgitation; HF, Heart Failure; MR, Mitral Regurgitation; NS, Not Significant; NYHA, New York Heart Association; PY, Person-Years; TEER, Transcatheter Edge-to-Edge Repair.

## Data Availability

No new data were created or analyzed in this study. Data sharing is not applicable to this article.

## Abbreviations

AF	Atrial Fibrillation
A-SMR	Atrial Secondary Mitral Regurgitation
A-STR	Atrial Secondary Tricuspid Regurgitation
CKD	Chronic Kidney Disease
CMR	Cardiac Magnetic Resonance
CT	Computed Tomography
CTR	Cardiothoracic Ratio
CV	Cardiovascular
DMR	Degenerative Mitral Regurgitation
H2FPEF	H2FPEF Score (clinical score for HFpEF probability)
HF	Heart Failure
HFpEF	Heart Failure with Preserved Ejection Fraction
HR	Hazard Ratio
IPTW	Inverse Probability of Treatment Weighting
LA	Left Atrium
LAA	Left Atrial Appendage.
LAP	Left Atrial Plication.
LAVi	Left Atrial Volume Index
LV	Left Ventricle
LVEDD	Left Ventricular End-Diastolic Diameter
LVEF	Left Ventricular Ejection Fraction
LVESD	Left Ventricular End-Systolic Diameter
MR	Mitral Regurgitation
MRI	Magnetic Resonance Imaging
NYHA	New York Heart Association functional classification
PML	Posterior Mitral Leaflet
PVI	Pulmonary Vein Isolation
SGLT2	Sodium–Glucose coTransporter-2
sPAP	Systolic Pulmonary Artery Pressure
TEE	TransEsophageal Echocardiography
TTE	TransThoracic Echocardiography
TEER	Transcatheter Edge-To-Edge Repair
TR	Tricuspid Regurgitation
TV	Tricuspid Valve
V-SMR	Ventricular Secondary Mitral Regurgitation
